# The structure and formation of giant Marimo (*Aegagropila linnaei*) in Lake Akan, Japan

**DOI:** 10.1038/s41598-021-01028-5

**Published:** 2021-11-10

**Authors:** Keisuke Nakayama, Katsuaki Komai, Keisuke Ogata, Toshiro Yamada, Yukinobu Sato, Fumiya Sano, Shintarou Horii, Yuichiro Somiya, Etsuko Kumamoto, Yoichi Oyama

**Affiliations:** 1 Graduate School of Engineering, Kobe University, Kobe, 657-8501 Japan; 2 Faculty of Engineering, Kitami Institute of Technology, Kitami, 090-8507 Japan; 3 Nishimuragumi Co., Ltd., Yubetsu, 099-6404 Japan; 4 Housui Engineering Consultants Co., LTD., Sapporo, 065-0033 Japan; 5 Center of Radiology and Radiation Oncology, Kobe University Hospital, Kobe, Japan; 6 Marimo Research Center, Kushiro Board of Education, Kushiro, 085-0467 Japan

**Keywords:** Limnology, Civil engineering, Hydrology

## Abstract

*Aegagropila linnaei* is a freshwater green alga, which at one time was distributed widely in the northern hemisphere. The aggregate often forms beautiful spherical shapes known as “lake balls” or “Marimo”. The population of Marimo has been rapidly decreasing worldwide, and today the large Marimo, with a diameter of more than 12 cm, exit only in Lake Akan in Japan. However, how Marimo grow and maintain their unique spherical shape in natural habitats remains unsolved. Here we show that Marimo are “polished” into spheres by the rotation induced by wind waves. Such a process enhances the water exchange between the interior and exterior of the Marimo, thereby recycling nutrients for growth. Our results provide an intriguing model of a physical environment interacting with biological processes in a self-sustaining ecosystem. We also demonstrate that Marimo have a spherical annual ring structure, and their growth rate is associated with ice cover. The balance between the ecology of Marimo and the water environment in Lake Akan is highly vulnerable and at risk of irreversible degradation. We must endeavor to rescue Marimo from the fate of a "canary in the coal mine" of global climate change.

## Introduction

**Figure 1:**
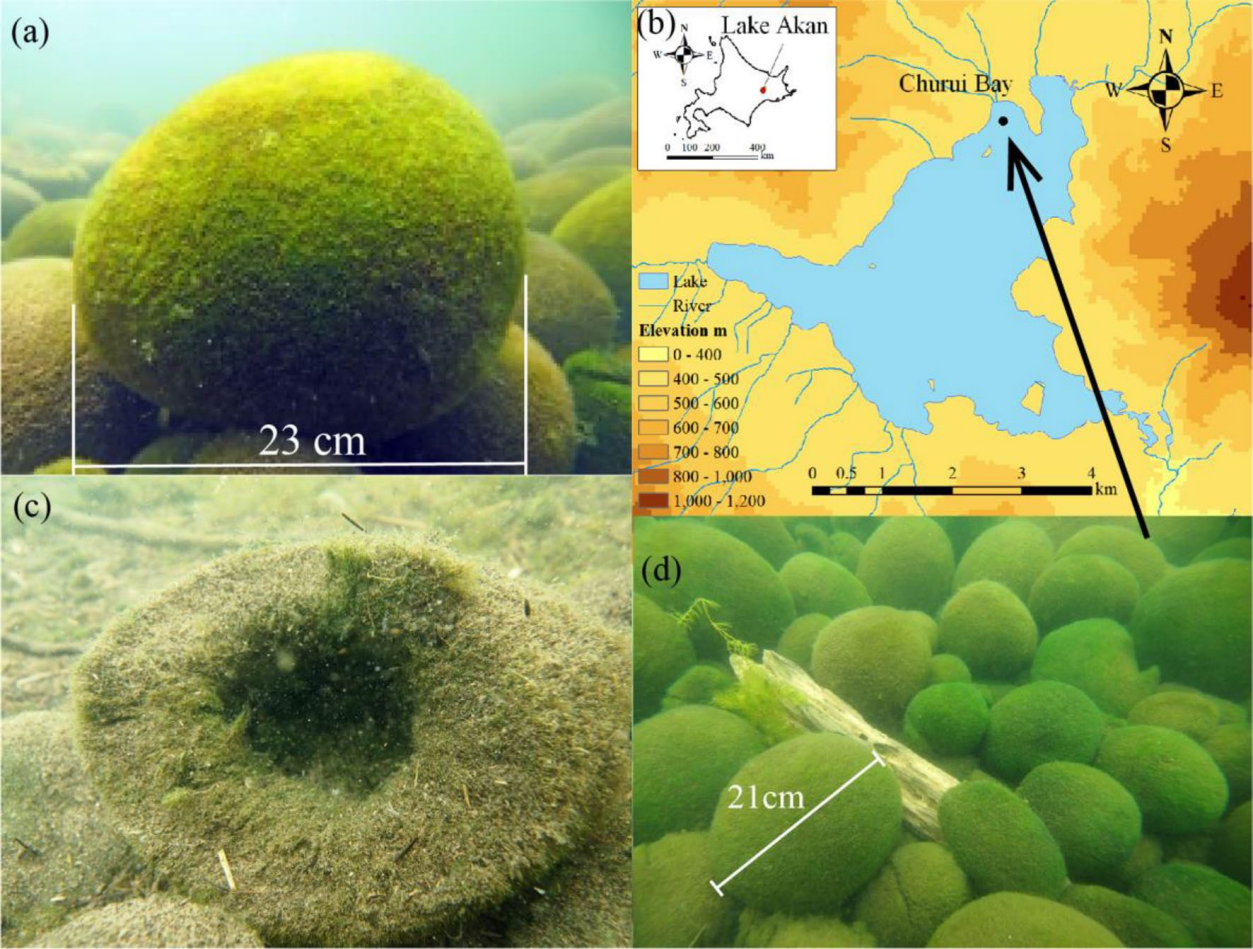
Marimo in Lake Akan. (**a**) Marimo, (**b**) Lake Akan and Churui Bay, (**c**) Collapsed Marimo, (**d**) Marimo colony in Churui Bay. Map made by ESRI ArcGIS 10.8.

*Aegagropila linnaei* is a freshwater green alga^1,2^, which at one time was distributed widely around the northern hemisphere^1^. *A. linnaei* has a branched filamentous form and grows as attached, floating unattached, and aggregated growth forms^3,4^. The aggregate often forms beautiful spherical shapes known as “lake balls,” “*Cladophora balls*,” or “Marimo (in Japanese),” which reach a size of over 20 cm in diameter^3–5^ (Fig. 1a). *A. linnaei* is thought to grow under multiple environmental factors, such as hydro-meteorological and topographical conditions^4,5,7,12–14^.

The population of *A. linnaei* has declined worldwide^6–11^. An investigation of *A. linnaei* in the Netherlands concluded that the species had disappeared in all regions of that country due to eutrophication^9^, and another study reported that large Marimo (10–12 cm in diameter or more) were found only in Lake Mývatn in Iceland and Lake Akan in Japan in 2004^7^. The Marimo population in Lake Mývatn started to decline with the increasing algae bloom around the 1990s and almost disappeared entirely in 2013^11^. Since then, the large Marimo, with a diameter of more than 12 cm, exit in Lake Akan only in the world. Marimo in Lake Akan were designated as a Natural Monument by the Japanese Government in 1921 and a Special Natural Monument in 1952. Nevertheless, two out of the four colonies of Marimo disappeared in the 1940s due to sediment deposition caused by lumber transport using rivers^15^. The other two colonies had also been at risk of extinction due to such factors as the water-drawdown cycle of hydropower plants, disturbance of the lake bottom by ships, and eutrophication^16–19^. Fortunately, these two colonies have been conserved until now and continue to produce giant Marimo (over 20 cm in diameter). An isozyme study showed that the spherical colonies in Lake Akan were genetically homogeneous, implying vulnerability to environmental stressors^20^. Another study found that Japan was the ancestral area for *A. linnaei* based on internal transcribed spacer (ITS) ribosomal DNA sequences^21^. Thus, Lake Akan is considered the only site with potential for the conservation of *A. linnaei*. However, how Marimo grows and maintains its unique spherical shape in natural habitats remains unsolved. Since the late nineteenth century it has been suggested that Marimo are formed by the rotation induced by wind waves^5,22–24^, meaning that hydrological studies are needed to clarify Marimo's formation in the lake. Nonetheless, there have been few studies of the physical characteristics of Marimo and their habitats, because instruments with a sufficient capacity for monitoring underwater dynamics were not widely available before the decline of the Marimo populations worldwide.

In 2014, the Japan broadcasting corporation took the first underwater footage of the rotation of Marimo in Lake Akan. By analyzing these video images and the accompanying meteorological data, our research team was able to verify that wind waves rotate Marimo^14,25^. In the six years since those experiments were conducted, further advances have been made in measurement technologies, and the new systems have the potential to provide additional data to unravel the age-old mystery of how Marimo grow and maintain their sizeable spherical shape. In the present study, therefore, we investigated the behavior of Marimo using underwater video cameras, an anemometer, and a wave height meter. First, we revealed the internal structure of Marimo by using magnetic resonance imaging. Second, the physical and meteorological parameters required for the formation of Marimo were obtained. Finally, we revealed that Marimo grow due to the interaction between physical and biological processes in a self-sustaining ecosystem by analyzing the exchange of dissolved oxygen (DO) and nutrients between the interior and exterior of Marimo.

### Marimo in Lake Akan

Lake Akan is a caldera lake in the Akan-Mashu National Park of the eastern Hokkaido Island with a surface area of 13.28 km^2^ and depth of 45 m. Marimo grow in Churui Bay and Kinetanpe Bay in the northern portion of Lake Akan (Fig. 1b). Giant Marimo grow in a water depth from 2 to 3 m, and collapsed Marimo and unattached floating mats exist in deeper water. Marimo of a few cm to about 30 cm diameter form layers, with the smallest spheres lying in the bottom layer, and the giant Marimo inhabiting the top layer (Fig. 1a,d). The individual Marimo consist of an aggregation of branched uniseriate green alga filaments and grow by photosynthesis. Marimo of more than 10 cm have a central cavity with a surface thickness of 4–5 cm, which is the approximate depth limit that the *A. linnaei* can exploit for photosynthesis^26^ (Fig. 1c). In the collapsed Marimo shown in Fig. 1c, fine sediments, including particulate organic matter, cover the surface, suggesting that the Marimo must somehow shake or throw off these deposits by oscillating and rotating in order to sustain its growth by photosynthesis^14,27^. Note that the collapsed Marimo get into a few cm pieces; still, they survive in the colony and become a giant Marimo though the formation mechanisms remain unsolved.

**Figure 2:**
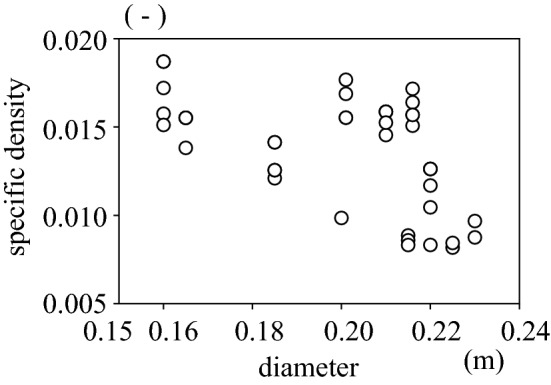
Diameter and underwater specific density of Marimo.

When the Marimo's diameter becomes greater than 10 cm, a central cavity appears inside due to decomposition, resulting in septic matter accumulation. Thus, the underwater specific density of Marimo becomes smaller with increasing diameter (Figs. 2, 6, 7, 8). For example, the specific density of a 23 cm diameter Marimo sphere is half that of a 16 cm diameter sphere. Marimo with small diameter are likely to drop into gaps in the layered colony structure, resulting in the predominance of large diameter Marimo at the top layer. Therefore, we identified an additional phenomenon occurring in this species: the oscillational motion of wind waves moves the giant Marimo to the top layer because of their smaller underwater specific density, as shown in Fig. 1a,d. Note that the gas bubbles due to photosynthesis also change the buoyancy of the Marimo^28^.

## Results and discussion

### Annual rings of Marimo

**Figure 3:**
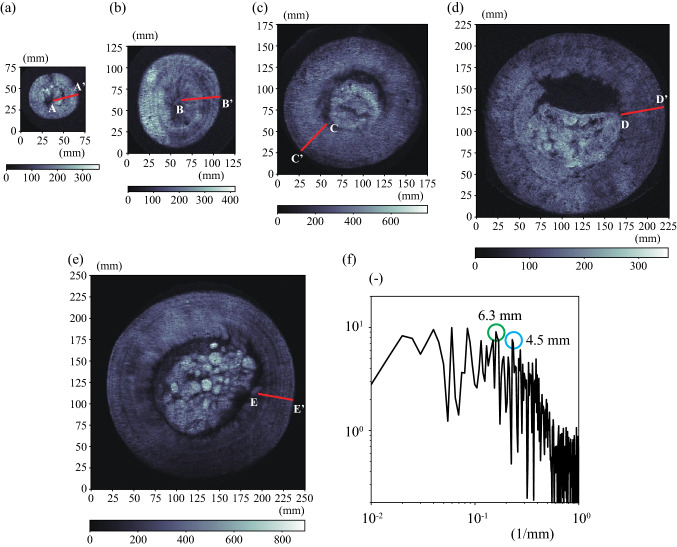
Magnetic resonance images. (**a**) Diameter of 6.0 cm, (**b**) Diameter of 10.0 cm, (**c**) Diameter of 16.0 cm, (**d**) Diameter of 20.0 cm, (**e**) Diameter of 22.0 cm, (**f**) Spectrum of MRI values along red lines.

We conducted a non-destructive analysis using magnetic resonance imaging (MRI) (Intera Achieva 1.5T Nova Dual; Philips) to investigate the void ratio inside of Marimo (Fig. 3). For MRI analysis of the void ratio, we placed Marimo into a polyethylene bag filled with water. We obtained a proton density-weighted image using Head Coil, which showed that the higher the density of green alga filaments, the larger the MRI value. The black area inside the Marimo sphere indicates the base of the individual Marimo, which is the origin of the Marimo and relocates to the center of the sphere when the Marimo grows sufficiently. We did not observe the base of the Marimo in the center of a 6 cm Marimo, suggesting that it was still growing (Fig. 3a). However, the base is located at the center in a 10 cm Marimo (Fig. 3b). In contrast, the central cavity's MRI values are higher than the other areas when the diameter is more than 16 cm (Fig. 3c–e). The higher central values may correspond to detached organic matter because of the lack of photosynthesis.

All Marimo had annual rings that appeared as sparse and dense patterns. The MRI values were confirmed to be relatively higher adjacent to the Marimo surface than a few mm inside. The MRI analysis was conducted using Marimo sampled in June and July, suggesting that the Marimo surface is highly dense in Spring and Summer compared to other seasons. Indeed, it was shown that spherical colonies might drift into shallow parts with gently sloping sandy bottoms, where, by the intensive rolling motion of the waves^5^. On the other hand, it was revealed that the aggregation of branched uniseriate green alga filaments gradually loosened without any external force and motion for ten years^23^. It was also reported that Marimo became bushy in a water tank with no movement^29^. Admittedly, there is no significant external force during winter because the water surface is entirely covered by more than 0.5 m thick ice. Therefore, the sparse and dense patterns may correspond to the annual change in the surface void ratio of Marimo. We calculated the spectrum using all MRI values along the normal direction to investigate the predominant thickness of the annual rings (Fig. 9). The maximum peak principal thickness was 6.3 mm, and the second peak was 4.5 mm (Fig. 3f).

### Rotation of Marimo

Inflow discharge into Churui Bay is about 0.2 m^3^/s and does not vary widely. Thus, the current induced by inflow is less than a few mm/s, suggesting that the rotation of Marimo is not influenced by an inflow effect. As a major external force, wind blowing over the water surface drives two different motions in a water body; one is the wind-driven current, and the other is the oscillational flow due to wind waves. However, the ice entirely covers the water surface in Lake Akan from December to April. Therefore, wind-driven currents or wind waves are the only factors controlling the oscillation and rotation of Marimo from May to November. It was shown that the wind-driven current washes giant Marimo from a colony to shore in the typhoon event, but does not occur on regular days^19^. In contrast, the oscillation due to wind waves is the dominant motion for Marimo on regular days^25^. They revealed that the fetch at Churui Bay is the longest, 2.5 km when the wind direction is from southeast to southwest, the prevailing wind direction in Lake Akan^24^. Therefore, even when the wind speed is not so high, such as the wind speed from 5 to 10 m/s, wind waves may induce enough oscillation to rotate Marimo^14^. We measured wind speed and wind direction at the height of 2 m above the water surface of Churui Bay in 2014 and 2015 (Fig. 10). The dominant wind cycle was 24 h, corresponding to a typical land and sea breeze in Lake Akan, resulting in a significant wave height of 0.2 m and a period of 1.5 s.

**Figure 4:**
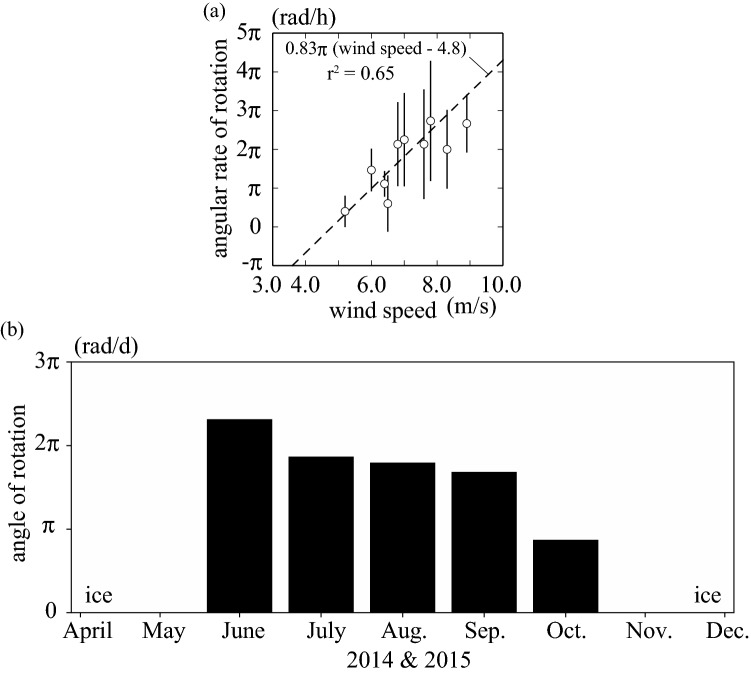
Wind and rotation of Marimo. (**a**) Wind speed and average angular rate of the rotation of Marimo. Solid lines indicate the standard deviation of average angular rate (average ± SD (n=3-10)). (**b**) The diurnal angle of the rotation of Marimo in 2014 and 2015.

To investigate how Marimo oscillate and rotate in response to wind waves, we recorded their response using an underwater video camera. The video images were recorded in 2014. Adequate wind waves are needed for Marimo to oscillate and rotate. For example, Marimo start rotating when the wind direction is from southeast to southwest. The diameter range was from 15 to 25 cm, and 12 Marimo were selected to investigate the relationship between wind speed and Marimo's rotational angular rate, which provided 52 data plots. First, we attempted to decide the critical wind speed at which Marimo start rotating using the wind speed and video images. 4.8 m/s was found to be the critical wind speed at which the Marimo began to rotate. Then, the average rotational angular rate was modelled as a function of wind speed, ${\text{r}}^{2} = 0.65$ ($p < 0.01$) (Fig. 4a). Note that it is needed to conduct more field observations and laboratory experiments to model Marimo's rotational angular rate by considering Marimo's diameter and how to overlap in the layers. The critical wind speed may change in a future study, and there is the possibility that the response of Marimo's rotational angular rate to the wind speed has an upper yield point. Therefore, we need to investigate Marimo's rotational angular rate by conducting more field observations. Additionally, other methods, like laboratory experiments, are necessary to clarify the relationship between wind waves and Marimo's rotational angular rate.

The monthly mean rotational angle was estimated by using wind data sets from June to October in 2014 and 2015 (Figs. 4b, 11). Then, the average rotational angular rate was modelled as a function of wind speed, r^2^=0.65 (p<0.01; a Shapiro-Wilk normality test and a Pearson correlation test using a statistical software R (ver.3.1.0) (Fig. 4a). The monthly mean rotational angle was largest in June and smallest in October. Consequently, the Marimo oscillate and rotate from June to October when the water surface is free from ice cover. Thus, the Marimo are polished due to the oscillation and rotation, enabling a highly dense surface of green alga filaments to form, and fixed into a ball by the rocking motion. On the other hand, since Lake Akan is covered by ice from December to April, the Marimo are in a hibernation-like state over this period due to low water temperature. As there is no external force to oscillate and rotate the Marimo, they do not become polished, suggesting that the surface density of green alga filaments becomes lower during the ice-cover period^23,29^. Thus, we conjecture that Marimo develop a sparse cover of green alga filaments each winter followed by a dense cover in summer, resulting in annual rings. As suggested above, the predominant thickness of the annual rings, composing Marimo, is 6.3 mm and 4.5 mm, using the spectrum's first and second peaks. Therefore, the regions of alternately sparse and dense green alga filaments enable us to estimate the Marimo's diameter growth rate, 12.6 mm/year or 9.0 mm/year. Here, we summarize the results as follows:

1. Inflow does not affect Marimo motion on regular days. In contrast, the strong wind, such as by typhoon, causes wind-driven current but not significant oscillational flow, resulting in that Marimo are washed from the colony to shore.
2. The fetch is a significant factor controlling wind waves in the Marimo colony, polishing the Marimo surface during no ice period^5,24,25^.
3. Our field observations revealed that land and sea breeze frequently occurs at Churui Bay, providing enough oscillational motion to Marimo during no ice period.
4. Marimo surface becomes bushy with no motion during the ice-cover period^29^.
5. During the ice-cover period, the water surface is covered entirely in Lake Akan, and the fetch becomes zero, meaning that there is no Marimo movement.
6. The MRI analysis revealed that the Marimo sampled in June and July have lower void ratio adjacent to the surface of Marimo than a few mm inside. Therefore, the sparse and dense patterns correspond to the annual growth rate of Marimo, suggesting that the Marimo's diameter growth rate may be 12.6 mm/year or 9.0 mm/year.

### Self-sustained growth of Marimo

**Figure 5:**
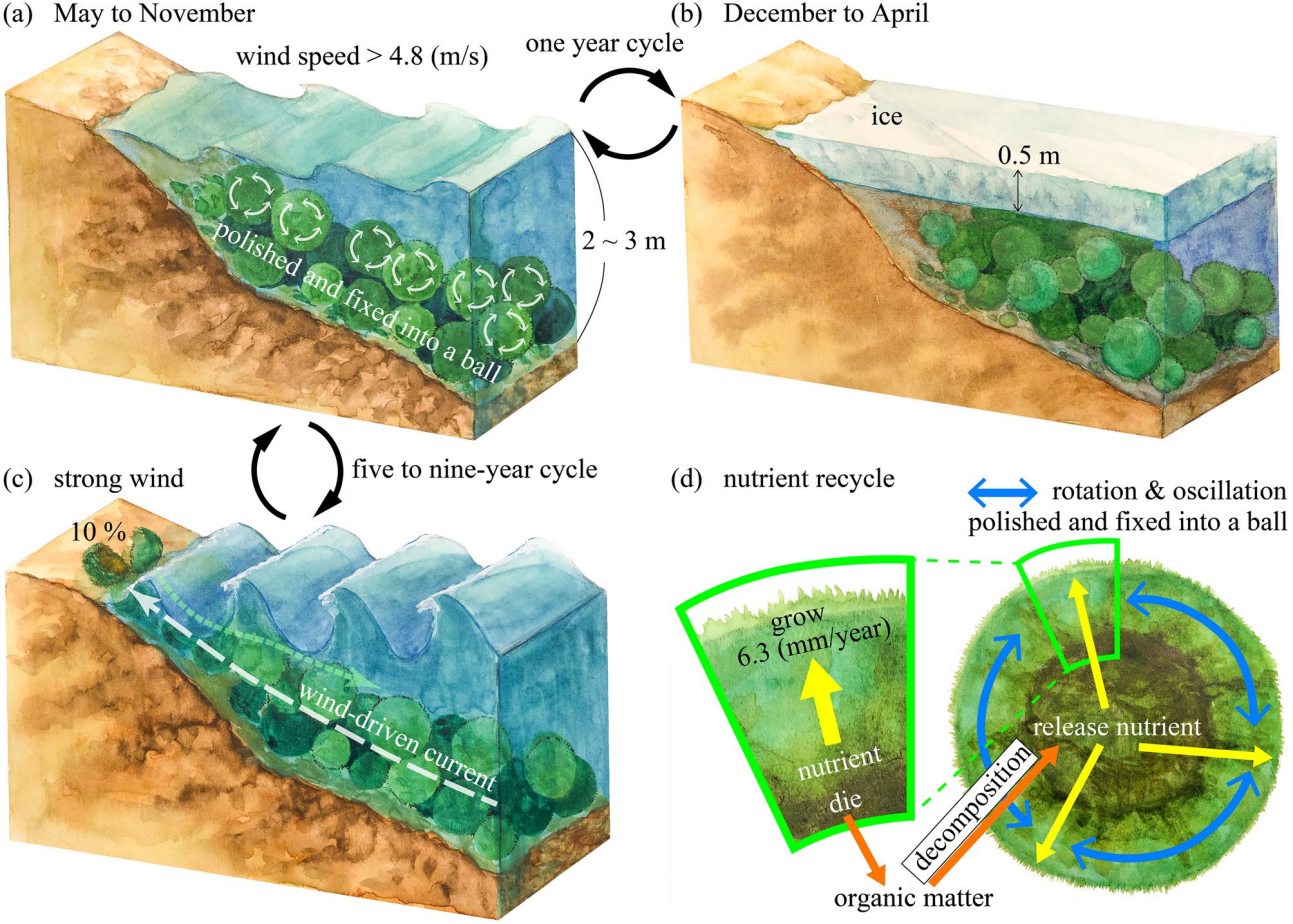
Formation of giant Marimo. (**a**) Growth period: June to October. (**b**) Hibernation period: November to May. (**c**) Marimo are washed ashore due to strong wind, and a small-size Marimo goes back to the Marimo colony. (**d**) Nutrient recycling in Marimo. Illustration adapted with permission from Reina Nakayama.

MRI analysis and field observations showed that the oscillation and rotation of Marimo due to wind waves polished and increased the density of green alga filaments at the surface of Marimo from June to October. In contrast, the void ratio became higher adjacent to the Marimo surface from December to April because the water surface was covered by ice, and there was no external force (Fig. 5a,b). In addition, the predominant growth rate in the diameter of Marimo was revealed to be 12.6 mm/year. Hence, it takes 17 years for Marimo to grow in diameter from 3 to 25 cm. In the event of a typhoon, strong winds often wash giant Marimo from a colony to shore. The giant Marimo in the shore collapse to the smaller size, tumble down over the sloping lake bottom and fall to lower layers in the colony (Fig. 5c). According to the Marimo Conservation Council^19^, such "generational change" occurs every 5 to 9 years^19^. The larger the diameter of the individual Marimo, the more likely it will be to lie on the colony's top layer. Since the occurrence cycle of most typhoons is shorter than the growth period of giant Marimo, generational change is one of the most significant factors controlling the formation of giant Marimo.

We conducted field observations to investigate the water quality inside and outside the Marimo by sampling 12 samples in June and August of 2015. The mean DO concentration in the Marimo interior was 7.8 mg/L with a mean water temperature of 11.5 °C in June. Also, the interior DO concentration was 2.3 mg/L with a mean water temperature of 27.5 °C in August, one of the hottest months in Lake Akan, but the interior water was not anoxic. Because the DO concentrations on the Marimo exterior in June and August were 10.5 mg/L and 8.7 mg/L, respectively, we anticipated that the water exchange might inhibit the occurrence of anoxia in the central cavity. We carried out laboratory experiments to evaluate the DO consumption rate of the interior water and then estimated the residence time of the interior water. The mean DO consumption rates in June and August were 0.67 mg/L/d and 1.9 mg/L/d, respectively. The mean residence time was found to be 105 h. On the other hand, detached organic matter decomposes in the Marimo interior, causing nutrients to be released, and thus we also measured total dissolved nitrogen (TDN) of 0.093 mg/L and total dissolved phosphorus (TDP) of 0.012 mg/L in the Marimo interior, which corresponded to the estimates in a previous study^30^. The exterior water's TDN and TDP were 0.008 mg/L and 0.005 mg/L, and Churui Bay is categorized as being in an oligotrophic state. The TDN/TDP values were 7.6 and 1.6 inside and outside Marimo, suggesting that high nutrients are released, condensed, and used to grow selectively. Therefore, the oscillation and rotation of Marimo play a significant role not only in polishing the Marimo and fixing it into a ball (Fig. 5a) but also in reusing the high levels of nutrients recycled inside the Marimo for growth (Fig. 5d).

## Conclusion

This study showed that a single organism, *A. linnaei,* existing in its aggregated form as a Marimo, is sustained by the world smallest nutrient recycling system, which is controlled by the oscillation and rotation of the Marimo due to wind waves. Namely, mortality, decomposition and mineralization managed by the oscillation and rotation of the Marimo enable the condensing of nutrients in the Marimo interior with an adequate balance of nitrogen/phosphorous. Thus, Marimo's green alga filaments grow by consuming oligotrophic nutrient on their exterior surfaces and by consuming high levels of nutrients recycled from the detached organic matter in the Marimo interior. The nutrient recycling system, which may be the world's smallest nutrient cycle, supported by the decomposition and mineralization of organic matter, is similar to the nutrient cycle in Shiretoko, the World Natural Heritage, in which salmon return to the rivers for spawning and transport nutrients from the ocean to the inland waters^31^. The nutrient cycle is one of the most important mechanisms to support an ecosystem in an oligotrophic natural environment. Unfortunately, the nutrient cycles of Marimo and other organisms might be in danger due to climate change. In Churui Bay, climate change may vary the mean wind speed and the frequency of strong wind, which would deteriorate the water exchange between the Marimo interior and exterior and the Marimo's growth rate. Since the self-sustained growth of Marimo is achieved by balancing the growth rate and generational change, the loss of an appropriate physical environment may cause irreversible degradation in the ecosystem for Marimo.

## Methods

### The underwater specific density of Marimo

**Figure 6:**
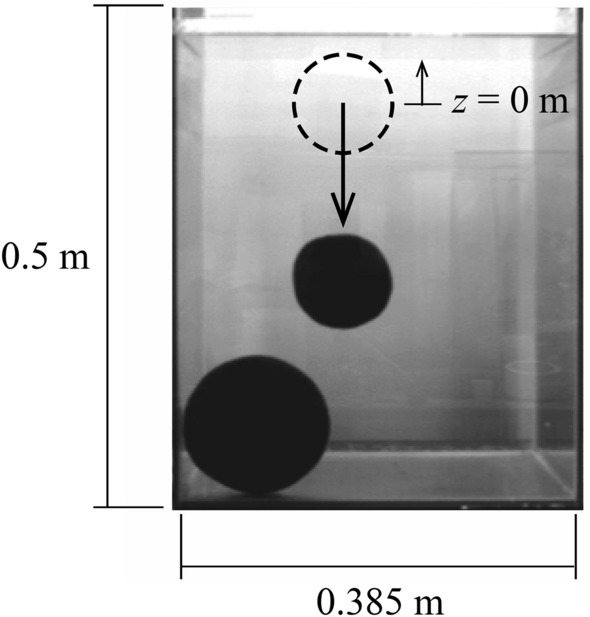
Underwater drop test of a Marimo to estimate its drag coefficient.

**Figure 7:**
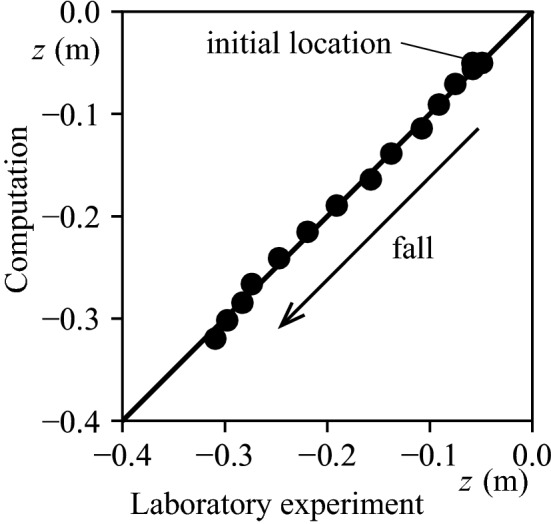
An example of an underwater drop test. Comparison of drop locations between the laboratory experiment and computation. The water surface corresponds to z = 0 m.

**Figure 8:**
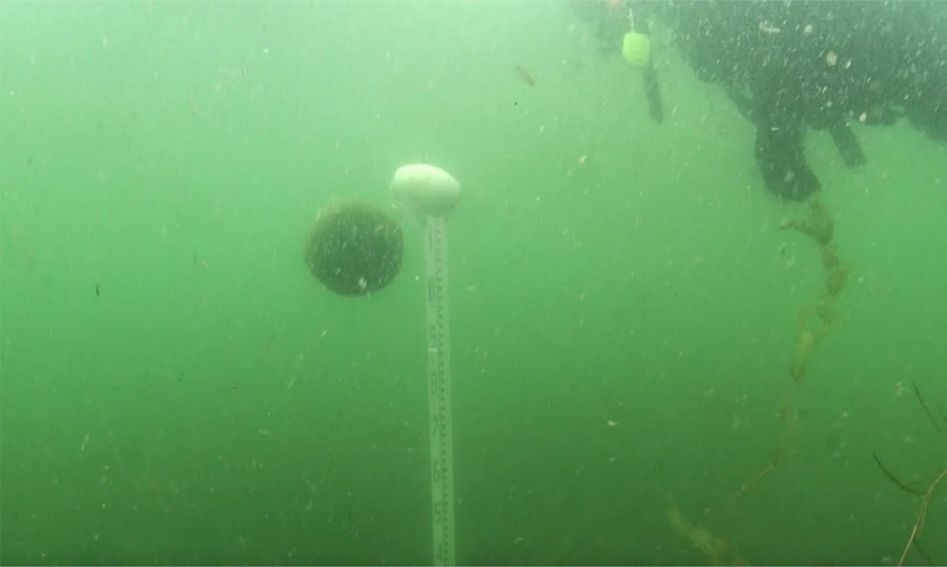
Field observation for estimating the underwater specific density of Marimo at Churui Bay in Lake Akan.

We conducted an underwater drop test using laboratory experiments and field observations to estimate Marimo's specific density ($\varepsilon = \left( {\rho_{m} - \rho_{w} } \right)/\rho_{m}$, $\rho_{m}$: the density of Marimo, $\rho_{w}$: the density of water). First, as the drag coefficient of Marimo is unknown, we carried out an underwater drop test using a water tank (Fig. 6). We used a water tank of 38.5 cm in width, 38.5 cm in length and 50.0 cm in height for the laboratory experiments. A high-speed camera with a frame rate of 125 s^−1^ was used to capture the central location of Marimo (Fig. 7). The drag coefficient of Marimo was revealed to be the same as a sphere, $C_{D} = 0.42$. Then, we conducted an underwater drop test in Churui Bay using 56 real Marimo on the 15th of June and 21st of August of 2015 (Fig. 8). To estimate the specific density of Marimo, we applied the vertical momentum equation, which revealed that the larger the Marimo's diameter, the smaller the Marimo's specific density:

$$V_{m} \frac{{\partial u_{m} }}{\partial t} = - \varepsilon gV_{m} - C_{D} A_{m} \left| {u_{m} } \right|u_{m} ,$$

where $e V_{m}$ is the volume of Marimo ($= 4\pi r_{m}^{3} /3$) (m^3^), $r_{m}$ is the radius of Marimo, $u_{m}$ is the vertical moving speed of Marimo (m/s), $\varepsilon$ is the underwater specific density of Marimo ($\varepsilon = \left( {\rho_{m} - \rho_{w} } \right)/\rho_{m}$), $\rho_{m}$ is the density of Marimo (kg/m^3^), $\rho_{w}$ is the density of water (kg/m^3^), $C_{D}$ is the drag coefficient of Marimo, and $A_{m}$ is the projected area of Marimo ($= \pi r_{m}^{2}$) (m^2^).

### Magnetic resonance image analysis

**Figure 9:**
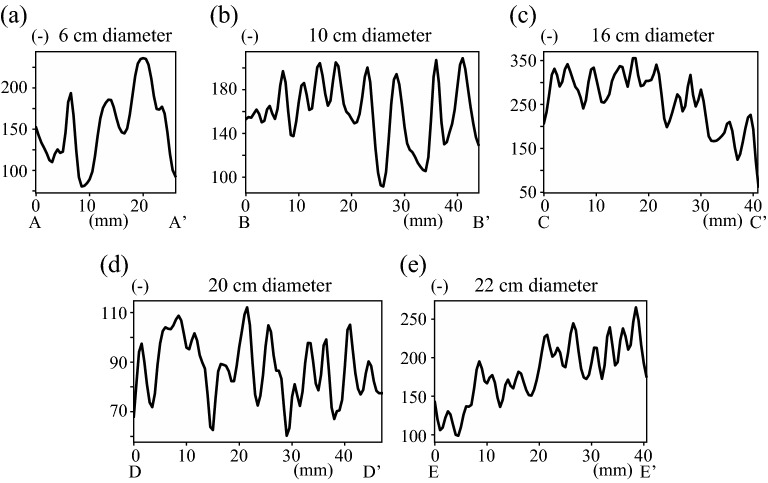
MRI values in Fig. 3. (**a**) A–A’, (**b**) B–B’, (**c**) C–C’, (**d**) D–D’, (**e**) E–E’.

We obtained a proton density-weighted image using Head Coil, which showed that the higher the density of green alga filaments, the larger the MRI value. Coronal images were obtained, and the conditions were TR/TE = 2700 ms/15 ms, TI (inversion time) = 1100 ms, NEX 2, acquisition matrix = 256 × 255, field of view = 256 mm × 256 mm, slice thickness = 4 mm, and image matrix = 512 × 512. MRI values along the lines of A–A', B–B', C–C', D–D' and E–E' in Fig. 3 are plotted in Fig. 9. The surface thickness is more than 4 cm and close to 5 cm in the Marimo of diameter 10 cm (B–B') and 20 cm (D–D'), and the sparse and dense green alga filament thickness ranges from 4 to 8 mm (Fig. 9b,d). In contrast, the minimum sparse and dense thickness is about 2 mm in the Marimo of diameter 22 cm (E–E'), but the dominant sparse and dense thickness is about 5 to 6 mm. The spectrum using all MRI values along the normal direction was obtained to investigate the predominant thickness of the annual rings (Fig. 9). In the spectrum analysis, all MRI values were connected into one continuous data after removing the trend. The spectrum analysis showed that the first peak growth rate was 12.6 mm/year, and the second peak growth rate was 9.0 mm/year. Figure 9 suggests that the growth rate of Marimo varies by the growth stage, environmental conditions, etc. It will be necessary to investigate the growth rate of Marimo in greater detail in a future study.

### Wind speed and direction

**Figure 10:**
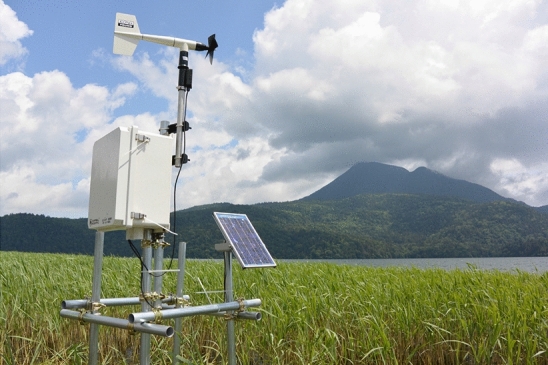
Meteorological station for measuring wind speed and direction at Churui Bay in Lake Akan.

**Figure 11:**
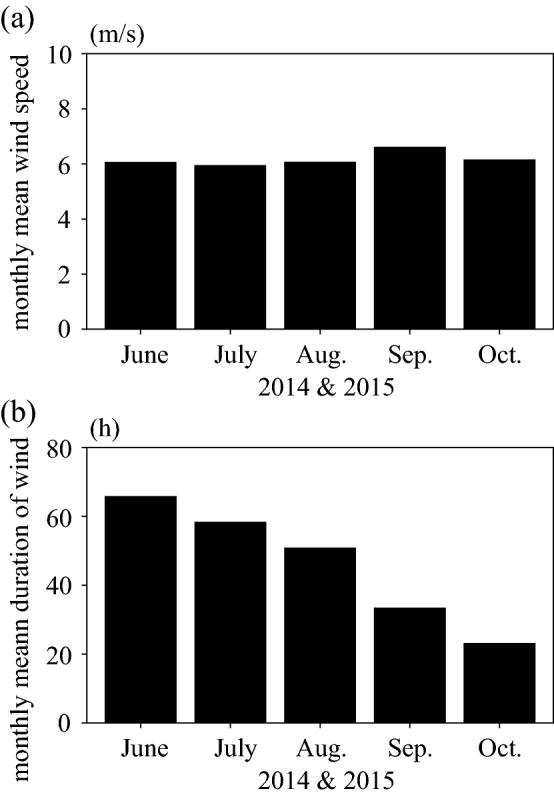
Wind speed and rotation of Marimo. (**a**) Monthly mean wind speed at Churui Bay in Lake Akan in 2014 and 2015. (**b**) Monthly mean duration of wind more than 4.8 m/s at Churui Bay in Lake Akan in 2014 and 2015.

Wind speed and direction were measured at 2 m above the water surface in Churui Bay from the 3rd of July to the 5th of November in 2014 and from the 11th of June to the 11th of August in 2015 (R. M. Young Company, Wind Monitor Model 05103) (Fig. 10). We applied the log law using the lake surface roughness to estimate wind speed at 10 m above the water surface. The roughness was 0.0001 m, and the wind speed at 10 m was 1.16 times greater than that at 2 m. Marimo moves predominantly when the wind direction is from the southeast to the southwest. The monthly mean wind speed was almost constant, about 6.0 m/s, from June to October in 2014 and 2015 (Fig. 11a). In contrast, the monthly mean duration of wind in June was the most prolonged, 60 h, and that in October was the shortest, 20 h (Fig. 11b). Significantly, Marimo showed the highest rate of oscillations and rotations soon after the ice at the water surface was melted. Note that the wind duration was summed up when wind speed was more than 4.8 m/s.

### Wave height and period

We conducted field observations to measure significant wave height and period at Churui Bay using a Wave Hunter set up at a water depth of 3 m (WH-503; I.O. Technique Co.). Wind waves were measured with an interval of 20 min from the 15th of July to the 14th of September in 2014, with an interval of 2 h from the 14th of September to the 5th of November in 2014, and with an interval of 2 h from the 8th of June to the 11th of August in 2015. Wilson's formulas were used to model significant wave height and period, which agreed well with the field observations. The appropriate wind duration was 30 min, and the 30 min mean significant wave height and period were used in the validation. The best fit fetch was from 2.0 to 2.5 km for Wilson's formulas^32^:

$$\frac{{gH_{1/3} }}{{U^{2} }} = 0.30\left[ {1 - \frac{1}{{\left\{ {1 + 0.004\left( {\frac{gF}{{U^{2} }}} \right)^{1/2} } \right\}^{2} }}} \right],$$

$$\frac{{gT_{1/3} }}{2\pi U} = 1.37\left[ {1 - \frac{1}{{\left\{ {1 + 0.008\left( {\frac{gF}{{U^{2} }}} \right)^{1/3} } \right\}^{5} }}} \right],$$

where $H_{1/3}$ is the significant wave height (m), $T_{1/3}$ is the significant wave period (s), $U$ is the wind speed (m s^−1^), $F$ is the fetch (m), and $g$ is the gravity acceleration (m s^−2^).

### Dissolved oxygen and consumption rate of dissolved oxygen inside of Marimo

We sampled the water inside Marimo using a syringe with a needle of diameter 0.69 mm and length 15 cm on the 9th of June and the 11th of August in 2015. Six syringe samples were collected for each Marimo to sample about 300 mL. Dissolved oxygen was measured using two portable water quality sensors (nos. HQ30d from Hach and 8,506,300 from Luminescent DO Sensor). The dissolved oxygen consumption rate was estimated by giving the initially saturated dissolved oxygen under constant water temperature conditions. Dissolved oxygen was measured every 3 h for 2 days. As a result, we obtained the following Arrhenius equation:

$$SOD = 1.02 \times 10^{8} \exp \left[ {\frac{{ - 7.43 \times 10^{ - 20} }}{{1.380649 \times 10^{ - 23} \left( {273.15 + T_{w} } \right)}}} \right],$$

where $T_{w}$ is the water temperature (°C).

We estimated the inside water's residence time using the following equation:

$$T_{R} = \frac{{{\text{DO}}_{out} - {\text{DO}}_{in} }}{SOD},$$

where $T_{R}$ is the residual time inside the Marimo (h), ${\text{DO}}_{out}$ is the DO around the Marimo (mg/L), ${\text{DO}}_{in}$ is the DO inside the Marimo (mg/L), and $SOD$ is the DO consumption rate inside the Marimo (mg/L/h).

### Nutrients

A diver sampled water inside the Marimo on the 31st of August in 2017; the same water sample was used for the dissolved oxygen measurement. 300 mL of water from the interior of the Marimo was collected, and the samples were brought to the laboratory in a cool box within a few hours. Water was filtered using a hydrophobic PTFE disposable membrane filter (DISMIC 25HP020AN; ADVANTEC), and TDN and TDP were measured using an autoanalyzer (QuAAtro 2HR; BLTEC).

## Data Availability

The data that support the findings of this study are available from the corresponding author upon reasonable request.
